# Novel Amino-Pyridine Functionalized Chitosan Quaternary Ammonium Derivatives: Design, Synthesis, and Antioxidant Activity

**DOI:** 10.3390/molecules22010156

**Published:** 2017-01-18

**Authors:** Qing Li, Caili Zhang, Wenqiang Tan, Guodong Gu, Zhanyong Guo

**Affiliations:** 1 Key Laboratory of Coastal Biology and Bioresource Utilization, Yantai Institute of Coastal Zone Research, Chinese Academy of Sciences, Yantai 264003, Shangdong, China; qli@yic.ac.cn (Q.L.); wqtan@yic.ac.cn (W.T.); 2 College of Chemistry, Chemical Engineering and Materials science, Shandong Normal University, Jinan 250014, Shandong, China; cafeapril@163.com; 3 University of Chinese Academy of Sciences, Beijing 100039, China; 4 Alliance Pharma, Inc., 17 Lee Boulevard, Malvern, PA 19355, USA; guguodong011@gmail.com

**Keywords:** antioxidant activity, chemical modification, chitosan derivatives, amino-pyridine

## Abstract

Chemical modification of chitosan is increasingly studied for its potential of providing new applications of chitosan. Here, a group of novel chitosan quaternary ammonium derivatives containing pyridine or amino-pyridine were designed and successfully synthesized through chemical modification of chitosan. Pyridine and amino-pyridine were used as functional groups to improve the antifungal activity of chitosan derivatives. The chitosan derivatives’ antioxidant activity against hydroxyl-radical and 1,1-Diphenyl-2-picrylhydrazyl (DPPH)-radical was tested in vitro. The results showed that chitosan derivatives had better water solubility and stronger antioxidant activity compared with chitosan in all assays. Especially, compounds **3C** and **3E** (with 3-amino pyridine and 2,3-diamino pyridine as substitute respectively) exhibited stronger hydroxyl-radical and DPPH-radical scavenging ability than other synthesized compounds. These data demonstrated that the synergistic effect of the amino group and pyridine would improve the antioxidant activity of chitosan derivatives, and the position of the amino group on pyridine could influence the antioxidant property of chitosan derivatives.

## 1. Introduction

As metabolic products of the human body, reactive oxygen species (ROS) are a great threat to health [1]. ROS, including hydroxyl radicals (•OH), hydrogen peroxide (H_2_O_2_), and superoxide anion (O_2_^•−^), are normally generated in the human body and scavenged by the antioxidant defenses system when ROS remains at physiological concentrations [2]. It was found that ROS concentrations in blood were strongly related to various pathological events such as aging, cellular injury, and DNA degradation. ROS can cause pathological damages such as cancer disease, diabetes, atherosclerosis, coronary heart disease, and many other diseases associated with aging to the organism, and lead to harmful alterations in foods and the pharmaceutical industry [3,4]. Meanwhile, the use of synthetic antioxidants, such as butylated hydroxyanisole, butylated hydroxytoluene, and propylgallate, has potential health hazards [5]. Therefore, it is of great interest among researchers to develop antioxidant supplements to help the human body reduce oxidative scratch and to search for natural antioxidants as alternatives to synthetic ones.

Currently, natural polysaccharides have attracted people’s attention for their unique physicochemical characteristics and bioactivity [6,7,8,9]. As one of the most abundant natural polysaccharides, chitosan and its derivatives are increasingly used in more and more fields, especially in the biomedicine area [10,11,12,13,14,15]. It is generally accepted that biological activities of polysaccharides are related with their molecular structure [16]. Therefore, chemical modification of polysaccharides is increasingly studied for its potential of providing new applications for such abundant polymers. Many chitosan derivatives were synthesized and their antioxidant activity was assessed accordingly. Our group worked on the antioxidant activities and antifungal activities of polysaccharides’ derivatives and proved that the chemical modification could improve the bioactivity of polysaccharides [9,17,18]. However, the application of chitosan and many chitosan derivatives is limited due to their solubility in aqueous solution. They are solvable mostly in diluted organic solutions such as formic acid, acetic acid, and succinic acid, as well as in very few inorganic solvents, such as hydrochloric, phosphoric, and nitric acid at pH below 6.5 [19]. Therefore, it is strongly desired that chitosan derivatives with high bioactivity and good water solubility are developed.

Based on several literature surveys, pyridine and its derivatives show a range of pharmacological activities, such as antibacterial, antitumor, antiparasite, and analgesic activity [20,21,22,23]. The high therapeutic property of pyridine-related drugs has encouraged medicinal chemists to synthesize a large number of chemotherapeutic agents [22]. Meanwhile, much literature has reported that saccharide derivatives containing amino groups have better antioxidant ability than natural saccharide [16,24]. Kim reported that 3-amino conjugates containing pyridine groups have good activity in vitro against most of the Gram-positive bacteria [25]. Hu proposed that the amino group at the C-4 position of pyridine ring should be an important factor that influences the antioxidant ability of the synthesized inulin derivatives [16].

On the basis of these observations, we thought of synthesizing a new class of chitosan derivatives, wherein amino-pyridine was linked to chitosan as a substituent. Our aim was to develop chitosan derivatives which possessed good antioxidant ability and water solubility. In this paper, we report the design, synthesis, and antioxidant ability of a group of novel chitosan derivatives. Firstly, the C_2_-NH_2_ was modified as a quaternary ammonium salt. The quaternary ammonium salt was selected by virtue of water solubility, which could enlarge the application of chitosan as a food preservative or bioactive matrix. Afterwards, the chloracetyl-*N*-trimethyl quaternary ammonium chitosan was synthesized by reaction between the C-6 hydroxyl of chitosan and chloracetyl chloride. Pyridine can be easily attacked by chlorine to give *N*-alkylpyridinium salts, as pyridine is a base with chemical properties similar to tertiary amines [26,27,28]. Besides, *N*-alkylpyridinium salts can also enable chitosan with better water solubility. Subsequently the amino-pyridine groups were introduced into chitosan through the reaction mentioned above. For comparison, a chitosan derivative with pyridine was also synthesized and studied under the identical conditions. The target chitosan derivatives designed in this way were expected to have advantageous features, namely high antioxidant activity and good water solubility. The chemical structures of the derivatives were characterized by Fourier Transform Infrared Spectroscopy (FT-IR) and ^13^C Nuclear magnetic resonance (^13^C-NMR). The antioxidant activities of chitosan and the synthesized chitosan derivatives were evaluated in vitro, and the relationship between the structure and the antioxidant activity of chitosan was discussed.

## 2. Results and Discussion

### 2.1. Chemical Syntheses and Characterization

**Scheme 1:**
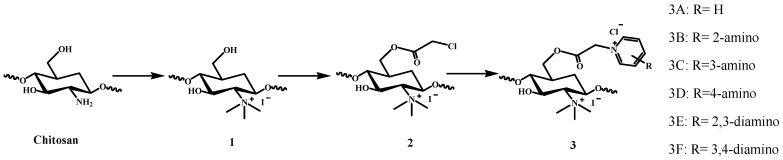
Synthetic scheme for the preparation of chitosan derivatives.

The synthetic procedures for the chitosan derivatives (compounds **3A**–**3F**) are shown in Scheme 1. *N*-trimethyl quaternary ammonium chitosan (**1**) was selected as an intermediate to protect the amino group of chitosan and prompt solubility of the synthesized chitosan derivatives. Chloracetyl quaternary ammonium chitosan (**2**) was then synthesized by reaction between the C-6 hydroxyl of chitosan and chloracetyl chloride. Chloracetyl quaternary ammonium chitosan is an excellent intermediate of the project as its chlorine can easily attack pyridine to give *N*-alkypyridinium salts [26,27,28]. The targeted chitosan derivatives (**3**) were synthesized through the nucleophile substitution reaction between chloracetyl quaternary ammonium chitosan (**2**) and pyridine or amino-pyridine.

**Figure 1:**
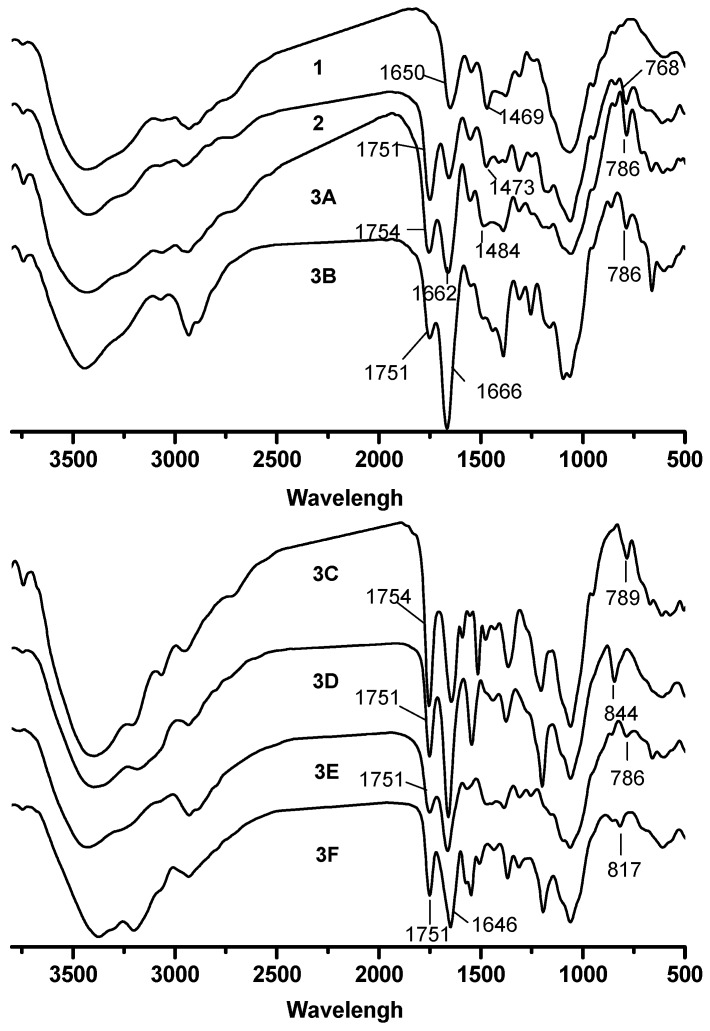
FT-IR spectra of intermediate products and the targeted chitosan derivatives.

**Figure 2:**
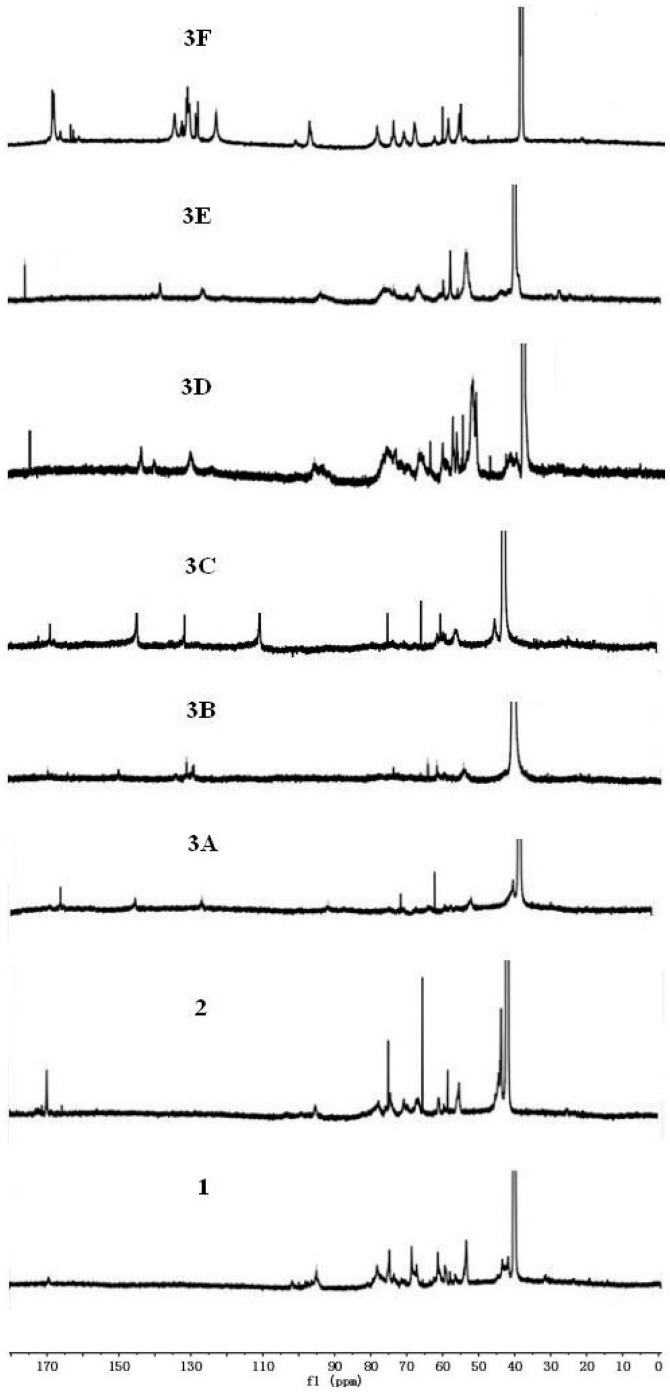
^13^C-NMR spectra of intermediate products and the targeted chitosan derivatives.

Each step of the synthesis was verified by FT-IR and ^13^C-NMR spectroscopy analyses. The FT-IR and ^13^C-NMR spectra of intermediate products, compounds **3A**–**3F**, are shown in Figure 1 and Figure 2 respectively. In the FT-IR spectrum of compound **1**, a new peak at 1469 cm^−1^ was ascribed to the characteristic absorption of N-CH_3_ [29]. In the ^13^C-NMR spectrum of compound **1** (Figure 2), the carbon of N-CH_3_ is clearly observed at 53.4 ppm. For compound **2**, the reaction of chloracetyl chloride with quaternary ammonium chitosan led to a new peak at 1751 cm^−1^, which could be attributed to carbonyl (C=O) [28]. Meanwhile, another new peak at 768 cm^−1^ was assigned to the C-Cl group [28]. In the ^13^C-NMR spectrum of compound **2**, the stretching vibration of the carbonyl group C=O at 167.9 ppm and that of quaternary ammonium at 53.8 ppm can be clearly observed (Figure 2). All of these characteristic peaks indicated that chloracetyl quaternary ammonium chitosan (compound **2**) was synthesized successfully.

As long as we get compound **2**, pyridine or amino-pyridine can be easily attacked by its chlorine to give *N*-alkylpyridinium salts, which will be used to synthesize the targeted chitosan derivatives. As shown in Figure 1, the peak at 768 cm^−1^ in the compound **2** spectrum disappears and a new peak appears at 786 which was assigned to the absorbance of pyridine in the spectrum of **3A**. In the spectra of compounds **3B**–**3F**, a prominent peak in the range of 1658–1666 cm^−1^ belongs to carbonyl (C=O), and the sorption band at about 786–844 cm^−1^ might be attributed to the pyridine ring of amino-pyridine. Another prominent peak in the range of 1546–1589 cm^−1^ belongs to the amino group, and the sorption band at about 1430–1484 cm^−1^ was ascribed to the characteristic absorption of N-CH_3_. As seen in Figure 2, the carbon signals in ^13^C-NMR spectra are well attributed to compounds **3A**–**3F**. The pyridine is clearly observed at 123.6–148.2 ppm as two new peaks. Meanwhile, the signals of C=O still exist at 167.5–174.6 ppm, and quaternary ammonium at 53.7–56.5 ppm can be clearly observed. All of those spectra indicated the successful synthesis of chitosan derivatives.

### 2.2. Antioxidant Activities

Chitosan has poor solubility in water, so we used water-soluble low molecular weight chitosan in the antioxidant activity test. Compounds **3A**–**3F** showed better water solubility than chitosan, and could be prepared as aqueous solution (0.1–1.0 mg/mL) at room temperature. The improved water solubility of chitosan derivatives should come from the quaternary ammonium salt at the C_2_ position of chitosan.

**Figure 3:**
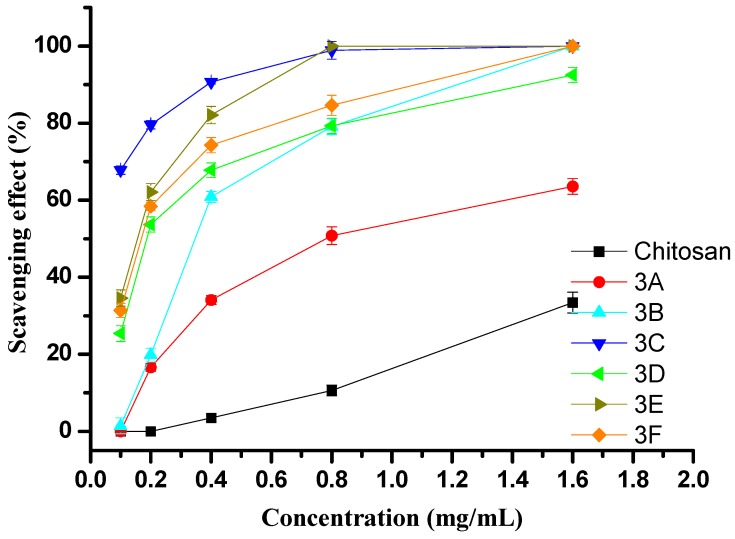
Hydroxyl-radical scavenging ability of chitosan and chitosan derivatives.

**Figure 4:**
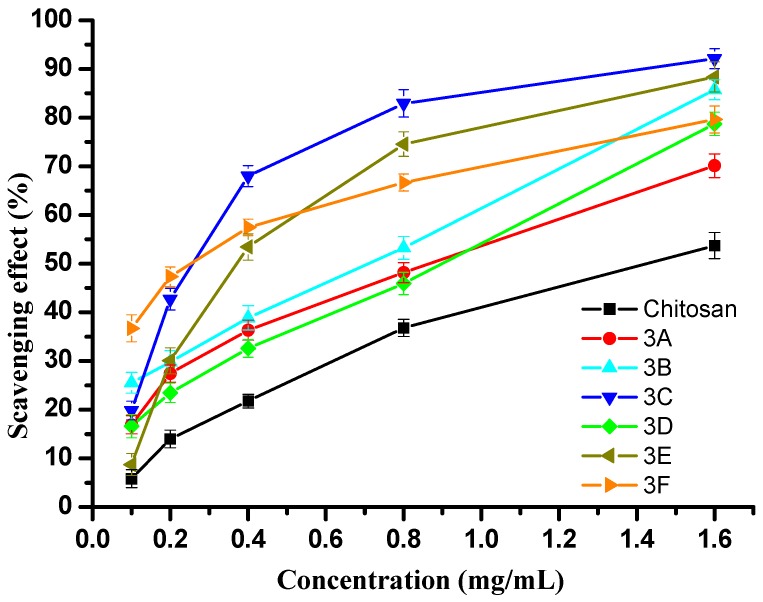
DPPH-radical scavenging ability of chitosan and chitosan derivatives.

Figure 3 and Figure 4 reveal the hydroxyl-radical scavenging ability and DPPH-radical scavenging ability of chitosan and compounds **3A**–**3F** at various concentrations, and the antioxidant activity results are shown in Table S1 as Supplementary Materials. As seen in Figure 3, the hydroxyl-radical scavenging ability of chitosan is weak, and compounds **3A**–**3F** give much stronger hydroxyl-radical scavenging ability compared with chitosan. Besides, the scavenging effect of all the samples mounts up with increasing concentration. It is obvious that compounds **3A**–**3F** show much better antioxidant activity due to the introduction of pyridine groups. Furthermore, it is apparent that compounds **3B**–**3F**, which have amino group at the periphery of molecular chains, have better hydroxyl-radical scavenging ability. This is possibly because of the presence of the more exposed amino group. The results above are in accord with the conclusion that aminated derivatives of saccharide are more potent than natural saccharide as a scavenger of hydroxyl radicals [16,24,30]. The scavenging property of chitosan and compounds **3A**–**3F** against DPPH-radical is shown in Figure 4. Similar to hydroxyl-radical scavenging ability, these compounds also possess more remarkable antioxidant activity, compared with chitosan. Furthermore, compounds **3B**–**3F** show better antioxidant activity at most concentrations. The antioxidant capacity observed in this experiment is consistent with the conclusion that amino groups can increase antioxidant activities [31]. Moreover, the results further confirm that the pyridine group and amino group grafted into compounds **3A**–**3F** contribute a lot to the antioxidant action and consequently increase the antioxidant activity of them.

As shown in Figure 3 and Figure 4, compounds **3C** and **3E** (with 3-amino pyridine and 2,3-diamino pyridine as substitute respectively) exhibit higher hydroxyl-radical and DPPH-radical scavenging ability than other synthesized compounds. Compounds **3C** and **3E** can scavenge the hydroxyl-radical completely at 0.8 mg/mL. These results indicate that the amino at the C-3 position of the pyridine ring will be more important than the amino at other positions on the scavenging activity against hydroxyl radicals and DPPH-radicals. The results may be illustrated by which oxidative stress can cause damage by stimulating the free radical chain reaction. Free radical chain reactions may be inhibited by adding preventive antioxidants that retard the formation of free radicals or stabilize free radicals. The electron cloud density of the 3-position of pyridine is higher than that of the 2- and the 4-position [32]. The amino group in pyridine may play an important role to act as an electron donor to quench free radicals by providing an electron, conceivably via an electron attack on the free radicals [5,33]. The stronger electron-donating groups tend to donate more electrons to quench more reactive free radicals, which may help stabilize the free radicals’ form [33,34]. Therefore, the stronger electron-donating groups show better influence on the antioxidant activity, while the weaker electron-donating groups exhibit moderate effects. Based on our experiment results and literature mentioned above, it will be reasonable to presume that the position of the amino group on pyridine can influence the antioxidant property of saccharide derivatives. Further comprehensive investigation to ascertain this hypothesis on the antioxidant and structure–activity relationship will be carried out in a future study.

## 3. Experimental Section

### 3.1. Materials

Chitosan was purchased from Qingdao Baicheng Biochemical Corp. (Qingdao, China). Its degree of deacetylation is 97% and the viscosity-average molecular weight is 7.0 × 10^4^. The 2-Aminopyridine, 3-aminopyridine, 4-aminopyridine, 2,3-diaminopyridine, and 3,4-diaminopyridine were purchased from Sigma-Aldrich with a minimum purity of 98%. The other reagents such as iodomethane, sodium iodide, sodium hydroxide, chloracetyl chloride, pyridine and solvents are analytical grade and were supplied by Sinopharm Chemical Reagent Co., Ltd., Shanghai, China.

### 3.2. Analytical Methods

FT-IR spectra were measured on a Jasco-4100 Fourier Transform Infrared Spectroscopy (Japan, provided by JASCO Co., Ltd., Shanghai, China) with KBr disks. ^13^C Nuclear Magnetic Resonance (^13^C-NMR) spectra were measured with a Bruker AVIII-850 Spectroscopy with TCI Cryo Probe (Switzerland, provided by Bruker Tech. and Serv. Co., Ltd. Beijing, China). The elemental analyses (C, H, and N) were performed on a Vario Micro Elemental Analyzer (Elementar, Langenselbold, Germany). The Degree of Substitution (DS) was calculated based on elemental analysis results.

### 3.3. The Synthesis of Chitosan Derivatives

The synthetic routes for the preparation of chitosan derivatives are shown in Scheme 1.

Preparation of *N*-trimethyl quaternary ammonium chitosan (compound **1**) [35]: a mixture of Chitosan (0.65 g, 4.0 mmol), 2.23 g sodium iodide, 4 mL aqueous sodium hydroxide solution (15%, *w*/*v*), and 4 mL iodomethane in 30 mL *N*-methyl pyrrolidone (NMP)was stirred at 60 °C for 1 h. The mixture was precipitated into ethanol, and the precipitate was collected by filtration and washed by ethanol. The products were dried at 60 °C for 24 h, yield: 75.0%; DS_quaternary_ 0.91; ^13^C-NMR/DMSO: δ 95.1–56.6 ppm (pyranose rings); δ53.4 ppm (carbon of quaternary ammonium); FT-IR (thin film): *v* 3436 (NH_2_ and OH), *v* 1469 (C-H of quaternary ammonium).

The 6-Chloracetyl-*N*-trimethyl quaternary ammonium chitosan (compound **2**) [36]: 042 g (1.25 mmol) quaternary ammonium chitosan (compound **1**) was dissolved in 15 mL DMSO at room temperature, and 0.2 mL chloracetyl chloride was added drop wise. After stirring for 12 h at room temperature, the mixture was precipitated into acetone, and the precipitate was collected by filtration and washed by acetone. The product was dried through freeze drying, yield: 68.0%; ^13^C-NMR/DMSO: δ167.9 ppm (carbon of carbonyl); δ93.4–56.7 ppm (pyranose rings); δ 53.8 ppm (carbon of quaternary ammonium); FT-IR (thin film): *v* 3424 (NH_2_ and OH), *v* 1751 (C=O of chloracetyl), *v* 1473 (C-H of quaternary ammonium), *v* 768 (C-Cl of chloracetyl).

Preparation of compounds **3A**–**3F** [16,37]: a solution of compound 2 (5 mmol) and pyridine or amino-pyridines (15 mmol) in 100 mL DMF was stirred for 24 h at 60 °C. The solutions were precipitated with excess acetone and filtered, washed carefully with acetone. The unreacted amino-pyridines and other byproducts were extracted in a Soxhlet apparatus with acetone for two days. The products were obtained by freeze drying.

**3A**: yield: 66.0%; DS: 0.82; ^13^C-NMR/DMSO: δ 167.5 ppm (carbon of carbonyl); δ 146.9 and 128.3 ppm (pyridine rings); δ 93.1–53.5 ppm (pyranose rings); δ 54.1 ppm (carbon of quaternary ammonium); FT-IR (thin film): *v* 3428 (NH_2_ and OH), *v* 1754 (C=O of chloracetyl), *v* 1484 (C-H of quaternary ammonium), *v* 786 (C-H of pyridine).

**3B**: yield: 60.0%; DS: 0.74; ^13^C-NMR/DMSO: δ 168.4 ppm (carbon of carbonyl); δ 149.0 and 130.7 ppm (pyridine rings); δ 93.5–58.5 ppm (pyranose rings); δ 53.7 ppm (carbon of quaternary ammonium); FT-IR (thin film): *v* 3444 (NH_2_ and OH), *v* 1751 (C=O of chloracetyl), *v* 1442 (C-H of quaternary ammonium), *v* 786 (C-H of pyridine).

**3C**: yield: 83.4%; DS: 0.73; ^13^C-NMR/DMSO: δ 169.2 ppm (carbon of carbonyl); δ 148.2 and 132.3 ppm (pyridine rings); δ 106.9–58.5 ppm (pyranose rings); δ 53.4 ppm (carbon of quaternary ammonium); FT-IR (thin film): *v* 3394 (NH_2_ and OH), *v* 1754 (C=O of chloracetyl), *v* 1430 (C-H of quaternary ammonium), *v* 789 (C-H of pyridine).

**3D**: yield: 84.0%; DS: 0.82; ^13^C-NMR/DMSO: δ 174.6 ppm (carbon of carbonyl); δ 143.9 and 130.9 ppm (pyridine rings); δ 96.9–58.3 ppm (pyranose rings); δ 56.4 ppm (carbon of quaternary ammonium); FT-IR (thin film): *v* 3397 (NH_2_ and OH), *v* 1751 (C=O of chloracetyl), *v* 1442 (C-H of quaternary ammonium), *v* 844(C-H of pyridine).

**3E**: yield: 73.0%; DS: 0.72; ^13^C-NMR/DMSO: δ 174.3 ppm (carbon of carbonyl); δ 143.1 and 130.8 ppm (pyridine rings); δ 96.2–58.8 ppm (pyranose rings); δ 54.1 ppm (carbon of quaternary ammonium); FT-IR (thin film): *v* 3428(NH_2_ and OH), *v* 1751 (C=O of chloracetyl); *v* 1469 (C-H of quaternary ammonium), *v* 786 (C-H of pyridine).

**3F**: yield: 94.0%; DS: 0.76; ^13^C-NMR/DMSO: δ 168.6 ppm (carbon of carbonyl); δ 135.3 and 123.6 ppm (pyridine rings); δ 97.6–59.8 ppm (pyranose rings); δ 56.5 ppm (carbon of quaternary ammonium); FT-IR (thin film): *v* 3374 (NH_2_ and OH), *v* 1751 (C=O of chloracetyl), *v* 1434 (C-H of quaternary ammonium), *v* 817 (C-H of pyridine).

### 3.4. Antioxidant Ability

#### 3.4.1. Hydroxyl-Radical Scavenging Ability Assay

The test of hydroxyl-radical scavenging ability was carried out according to Liu’s methods with minor modification [4]. The reaction mixture, a total volume 4.5 mL, containing the samples of chitosan or chitosan derivatives **3A**–**3F** (10 mg/mL, 0.045, 0.09, 0.18, 0.36, and 0.72 mL), were incubated with EDTA-Fe^2+^ (220 μM), safranine O (0.23 μM), and H_2_O_2_ (60 μM) in potassium phosphate buffer (150 mM, pH 7.4) for 30 min at 37 °C. The absorbance of the mixture was measured at 520 nm. In the blank, samples were substituted with distilled water. Meanwhile, in the control, H_2_O_2_ was substituted with potassium phosphate buffer. Three replicates for each sample concentration were tested. The capability of scavenging hydroxyl radicals of the products was computed using the following equation:

Scavenging effect (%) = [(A_sample 520nm_ − A_blank 520nm_)/(A_control 520nm_ − A_blank 520nm_)] × 100

where A_blank 520nm_ is the absorbance of the blank at 520 nm; A_sample 520nm_ is the absorbance of the sample at 520 nm; A_control 520nm_ is the absorbance of the control at 520 nm.

#### 3.4.2. DPPH-Radical Scavenging Ability Assay

The 1,1-diphenyl-2-picrylhydrazyl (DPPH^•−^) scavenging property of the products was evaluated by the following method [12]: testing samples (10 mg/mL, 0.03, 0.06, 0.12, 0.24, and 0.48 mL) and 2 mL ethanol solution of DPPH (180 μM) were incubated for 30 min at 25 °C. Then, the absorbance of the remained DPPH-radical was measured at 517 nm against a blank. In the blank, samples were substituted with distilled water. Meanwhile, in the control, DPPH was substituted with ethanol. Three replicates for each sample concentration were tested and the scavenging effect was obtained according to the following equation:

Scavenging effect (%) = [(1 − (A_sample 517nm_ − A_control 517nm_)/A_blank 517nm_] × 100

where A_control 517nm_ is the absorbance of the control at 517 nm and A_blank 517nm_ is the absorbance of the blank at 517 nm.

All data were expressed as means ± standard deviation (SD). Data were analyzed by an analysis of variance (*p* < 0.05) to guarantee statistical significance and the means were separated by Duncan’s multiple range test. The results were processed by computer programs: Origin and Statistic software SPSS.

## 4. Conclusions

In this paper, a group of novel chitosan quaternary ammonium derivatives containing pyridine or amino-pyridine were successfully synthesized through chemical modification of chitosan. The antioxidant activity of chitosan and the synthesized chitosan derivatives were tested. It was found that the synthesized chitosan derivatives had better water solubility and stronger antioxidant activity compared with chitosan in all assays. These data demonstrate that the pyridine functional group can contribute to the biological activity against hydroxyl-radical and DPPH-radical. Moreover, the antioxidant activity of the synthesized chitosan derivatives, which had an amino group at the periphery of polymers, showed better antioxidant activity. This suggests that the synergistic effect of the amino group and pyridine will improve the antioxidant activity of chitosan derivatives. Furthermore, it will be reasonable to presume that the position of the amino group on pyridine can influence the antioxidant property of these chitosan derivatives. Further comprehensive investigation to ascertain this hypothesis on the antioxidant and structure–activity relationship will be carried out.

## Supplementary Materials

The following are available online at: http://www.mdpi.com/1420-3049/22/1/156/s1. Table S1: The measurements of antioxidant activity for chitosan and chitosan derivatives.

## Abbreviations

The following abbreviations are used in this manuscript:

ROS	Reactive oxygen species
DPPH	1,1-Diphenyl-2-picrylhydrazyl
DMSO	Dimethyl Sulphoxide
DMF	*N*,*N*-Dimethylformamide
EDTA	Ethylenediaminetetraacetic acid
NMP	*N*-Methyl pyrrolidone
