# Supplementary material for: Novel Amino-Pyridine Functionalized Chitosan Quaternary Ammonium Derivatives: Design, Synthesis, and Antioxidant Activity
Source: Molecules. 2017 Jan 18;22(1):156. doi: 10.3390/molecules22010156 (PMC6155944; doi:10.3390/molecules22010156)
Supplement: Supplementary file 1 [file molecules-22-00156-s001.pdf]

Supplementary Materials: Novel Amino-Pyridine Functionalized Chitosan Quaternary Ammonium Derivatives: Design, Synthesis, and Antioxidant Activity

Qing Li, Caili Zhang, Wenqiang Tan, Guodong Gu and Zhanyong Guo

Table S1. The measurements of antioxidant activity for chitosan and chitosan derivatives.

ROS (Concentration (mg/mL))		Scavenging Effect (%, Mean ± SD)						
		**Chitosan**	**3A**	**3B**	**3C**	**3D**	**3E**	**3F**
Hydroxyl-radical	0.1	0	0	1.4 ± 2.05	67.8 ± 1.06	25.4 ± 2.05	34.6 ± 2.12	31.4 ± 1.83
	0.2	0	16.6 ± 1.02	19.8 ± 1.75	79.6 ± 1.06	53.7 ± 1.97	62.1 ± 2.19	58.4 ± 1.06
	0.4	3.5 ± 0.99	34.1 ± 1.13	60.9 ± 1.41	90.7 ± 0.56	67.8 ± 1.91	82.1 ± 2.26	74.3 ± 1.97
	0.8	10.6 ± 1.14	50.8 ± 2.27	79.1 ± 2.05	98.9 ± 2.26	79.3 ± 1.97	100	84.6 ± 2.62
	1.6	33.4 ± 2.69	63.6 ± 2.05	100	100	92.5 ± 1.96	100	100
DPPH-radical	0.1	5.82 ± 1.85	16.9 ± 1.86	25.5 ± 2.11	19.8 ± 1.93	16.6 ± 2.37	8.7 ± 2.25	36.7 ± 2.77
	0.2	13.9 ± 1.83	27.4 ± 1.77	29.7 ± 2.36	42.7 ± 2.21	23.4 ± 2.01	30.1 ± 2.64	47.3 ± 2.0
	0.4	21.8 ± 1.40	36.3 ± 2.05	38.8 ± 2.49	67.9 ± 2.18	32.5 ± 1.08	53.4 ± 2.71	57.4 ± 1.71
	0.8	36.8 ± 17.6	48.1 ± 2.03	53.2 ± 2.35	82.9 ± 2.81	45.9 ± 2.26	74.6 ± 2.50	66.7 ± 1.70
	1.6	53.7 ± 2.69	70.1 ± 2.41	85.7 ± 2.06	92.1 ± 2.06	78.6 ± 2.41	88.4 ± 3.12	79.6 ± 2.76
